# Two-Step Source Tracing Strategy of *Yersinia pestis* and Its Historical Epidemiology in a Specific Region

**DOI:** 10.1371/journal.pone.0085374

**Published:** 2014-01-09

**Authors:** Yanfeng Yan, Hu Wang, Dongfang Li, Xianwei Yang, Zuyun Wang, Zhizhen Qi, Qingwen Zhang, Baizhong Cui, Zhaobiao Guo, Chang Yu, Jun Wang, Jian Wang, Guangming Liu, Yajun Song, Yingrui Li, Yujun Cui, Ruifu Yang

**Affiliations:** 1 State Key Laboratory of Pathogen and Biosecurity, Beijing Institute of Microbiology and Epidemiology, Beijing, China; 2 Qinghai Institute for Endemic Diseases Prevention and Control, Xining, China; 3 BGI-Shenzhen, Shenzhen, China; 4 School of Computer Science, National University of Defense Technology, Changsha, China; University of Helsinki, Finland

## Abstract

Source tracing of pathogens is critical for the control and prevention of infectious diseases. Genome sequencing by high throughput technologies is currently feasible and popular, leading to the burst of deciphered bacterial genome sequences. Utilizing the flooding genomic data for source tracing of pathogens in outbreaks is promising, and challenging as well. Here, we employed *Yersinia pestis* genomes from a plague outbreak at Xinghai county of China in 2009 as an example, to develop a simple two-step strategy for rapid source tracing of the outbreak. The first step was to define the phylogenetic position of the outbreak strains in a whole species tree, and the next step was to provide a detailed relationship across the outbreak strains and their suspected relatives. Through this strategy, we observed that the Xinghai plague outbreak was caused by *Y. pestis* that circulated in the local plague focus, where the majority of historical plague epidemics in the Qinghai-Tibet Plateau may originate from. The analytical strategy developed here will be of great help in fighting against the outbreaks of emerging infectious diseases, by pinpointing the source of pathogens rapidly with genomic epidemiological data and microbial forensics information.

## Introduction

Three pandemics caused by *Yersinia pestis* claimed hundreds of thousands of human lives and reshaped the history of human civilization [1], [2]. The major clinical forms of plague are bubonic, pneumonic, and septicemic. The pneumonic plague is the most threatening one because it is transmitted by respiratory droplets from person to person [3]. The natural plague foci of *Y. pestis* are still widely distributed in Asia, Africa, America, and Eastern Europe, and many new cases are reported to the World Health Organization each year [4]. A major concern about the plague is that *Y. pestis* strains are easily acquired from widely distributed natural foci and can be used for bioterrorism. Therefore, *Y. pestis* is classified by the U.S. Centers for Disease Control and Prevention as a Category A bioterrorism agent [5].

A critical step for handling a natural outbreak of infectious diseases or an outbreak caused by a bioterrorism attack is to identify the source of pathogens. Genetic variations between related microbial strains could be used to determine the origin, relationships, or transmission routes of pathogens in one outbreak [6], [7]. However, *Y. pestis* is genetically monomorphic [8]–[10]; thus, traditional molecular markers that target a few orthologous genes, such as 16S rDNA, and multi-locus sequence typing (which targets six to eight housekeeping genes) cannot provide enough resolution for tracing the source or inferring the spread patterns of this pathogen. Genome-wide markers based on comparative genomics, such as single nucleotide polymorphism (SNP), difference region (DFR), clustered regularly interspaced short palindromic repeats (CRISPRs), and multi-locus variable-number tandem repeat analysis (MLVA) [8], [11]–[15], have been used for screening a wide panel of isolates. However, the method based on DFR and CRISPRs still reveal relatively low discrimination power [12], [15]. The MLVA method can generate genotyping results with high resolution, but the phylogenetic relationship across samples is potential influenced by homoplasies caused by high mutation rates of some VNTR sites [16]–[18]. The SNPs identified from limited number of whole genome sequences would generate inevitable phylogenetic discovery bias in phylogenetic reconstruction and genotyping [19]. The flaws of these methods hamper the establishment of a robust and fully discriminatory phylogeny and affect the results of plague outbreak source tracing.

Whole genome sequencing (WGS) can provide the maximum resolution for genomic variations and has been used successfully in tracing the *Bacillus anthracis* strain that was used in the 2001 “Anthrax mail”” biocrimes [20], the Aum Shinrikyo cult's aerosolization of *B. anthracis* spores over Kameido, Japan [21], [22], and two New York bubonic plague cases [23]. The breakthroughs in massively parallel sequencing technology enable very high throughput sequence production within a short time at a relatively low cost, which improves the possibility of using WGS as a conventional method for microbial forensics and evolutionary analysis [24]–[27]. New sequencing technologies have been successfully employed to trace the source of nosocomial and community infections of methicillin-resistant *Staphylococcus aureus* [28], *Mycobacterium tuberculosis* [29], and *Klebsiella pneumonia* [30]. However, the large amount of WGS data imposes a heavy computational burden and hampers the ability to quickly acquire source tracing results during outbreaks of emerging infectious diseases.

In this report, we applied next-generation sequencing technology to determine the whole genome sequences of *Y. pestis* strains that were isolated from a primary pneumonic plague outbreak that occurred in 2009 in Xinghai, China [31]. We developed a two-step source tracing strategy to trace the origin of this outbreak and inferred the historical epidemic of the plague in the region.

## Results

### Two-step source tracing of the 2009 plague outbreak

**Figure 1:**
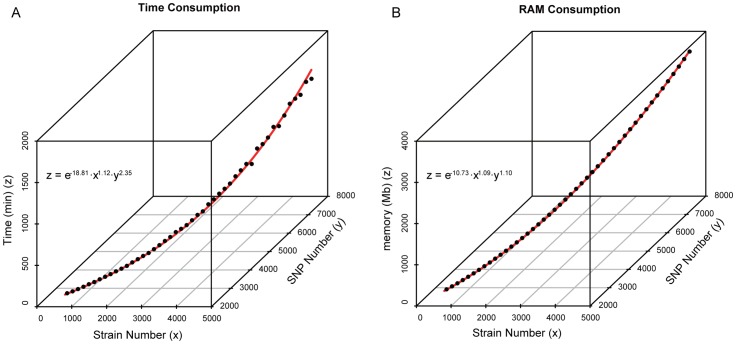
CPU running time and RAM consumption of phylogenetic reconstruction based on simulated mega dataset. The points represent the CPU running time (panel A) and RAM consumption (panel B) for reconstructing the MLTree based on different numbers of genomes and variations. The red curves represent the changes of both running time and RAM consumption, which follow the power function according to the formulas in the figure.

The phylogenetic analysis based on whole genome sequences usually consumes enormous amount of time and computational resources. Based on a simulated mega dataset, we estimated the time and computational cost for phylogenetic reconstruction. We found that the CPU running time and RAM consumption approximately followed the power function with increasing number of genomes and variations (Figure 1). Notably, when one genome was added into the simulated dataset, the genome included only one novel SNP site compared with the other existing genomes in the dataset, which represented the relatively low genetic diversity in the simulated population. It could be expected that more computational time and resources would be needed when handling the real data with higher diversity. Therefore, it is necessary to develop a simple analytical strategy to satisfy the urgent need for rapid source tracing during an infectious disease outbreak. Since the most important source tracing information is provided by the known close relatives of the outbreak strains, we only need to compare the genomes of outbreak strains to the genetically closest rather than to all available genomes. However, to get the information to which group of outbreak strains will be clustered into, we first need to compare the outbreak strains with the strain set representing the genetic and geographical diversities of the corresponding species. Accordingly, we developed a hierarchical two-step strategy, with the first step to define the phylogenetic position of the outbreak strains, and then the second step to get the detailed source tracing information by phylogenetic reconstruction of the outbreak strains, their close relatives, and historical strains in the outbreak region.

**Figure 2:**
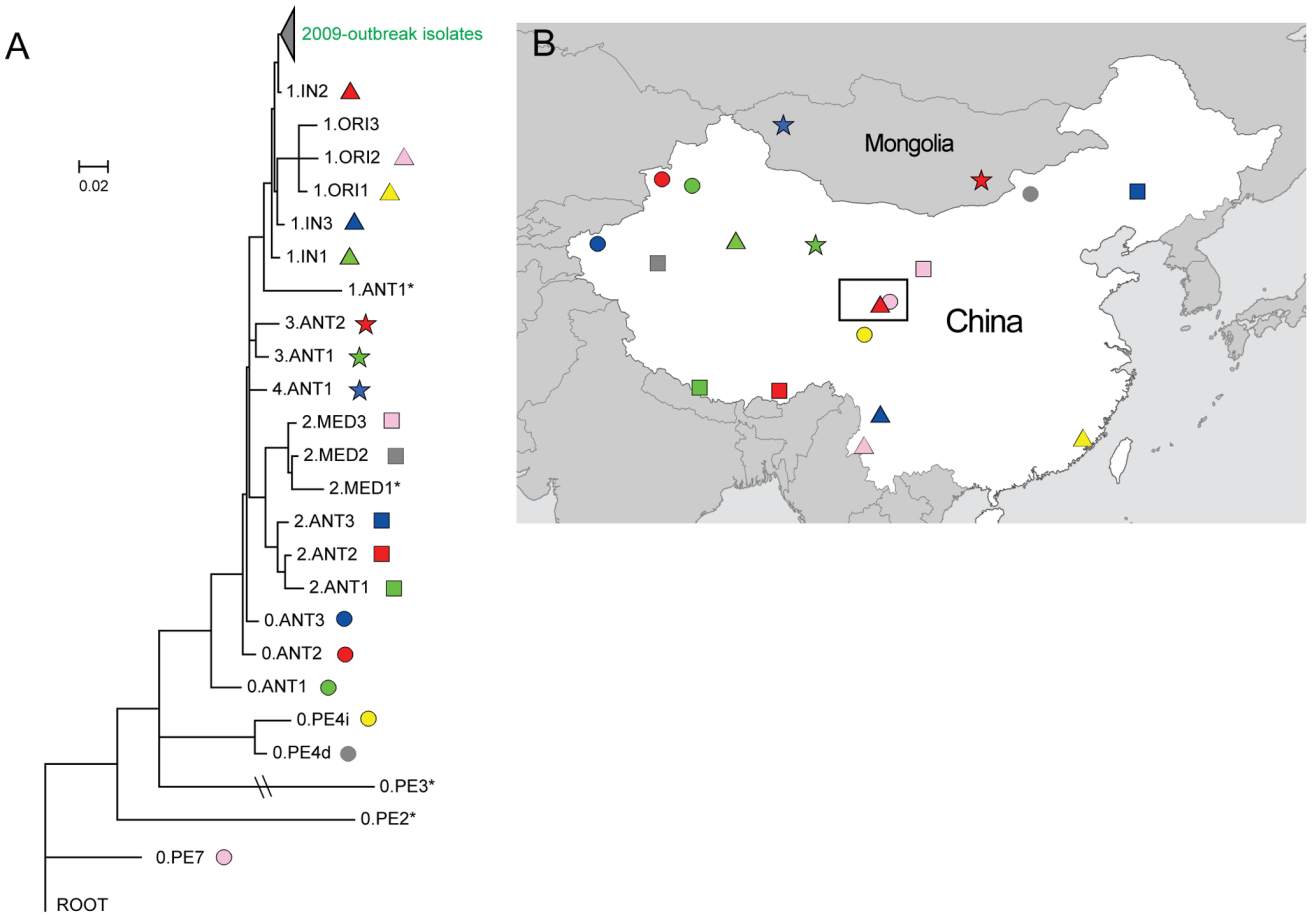
First step of source tracing: defining the phylogenetic position of outbreak strains. (A) MLTree of 2009 outbreak strains and 24 representative *Y. pestis* strains with global diversity. The branch length reveals genetic distance between isolates, except for the branch of 0.PE3, which was truncated for visual effect. The root was defined by the ancestor *Y. pseudotuberculosis*, IP32953. Each strain is labeled with different symbols and colors that correspond to its geographical distribution on the map in panel B. Strains that are not mapped in panel B are labeled with an asterisk. As no variations exist across outbreak strains, the corresponding branches were collapsed in the tree. All nodes have a bootstrap support of more than 90%. (B) Geographic information of the strains in the MLTree.

According to the phylogenomic analysis of more than 100 *Y. pestis* strains with global genetic variation, the population structure of this species included five main lineages (branches 0 to 4) and 23 phylogroups [32]. By using the newly developed strategy, we firstly selected one strain from each known phylogroup to represent the genetic polymorphism of the corresponding group, except for the 0.PE4 from which two strains were selected because of their distinct geographical distribution. Twenty-four strains, including 16 that were isolated from 12 natural plague foci of China and eight that were isolated from the former Soviet Union, Mongolia, Nepal, Kurdistan, and Africa (Table S1 and Figure 2), were selected to build the phylogenetic tree with seven outbreak strains. Based on 1,564 SNPs across these strains (Table S3), we employed three types of phylogenetic inference methods, including maximum likelihood, neighbor joining, and maximum parsimony, to reconstruct the phylogeny (Figures 2A and S1). All three methods yielded identical tree topologies with high bootstrap supports, thereby proving the robustness of the phylogenetic relationships.

According to the phylogenetic trees, no SNP variations exist among all seven outbreak isolates, including two isolated from the dead dogs and five from patients (Table 1) [31]. Based on the fact that the dog fed by the index patient first manifested the symptoms of the plague, the 2009 Xinghai outbreak could have resulted from a dog that was infected with *Y. pestis*, which was then transmitted to the index patient. This hypothesis is in agreement with previous conclusion from the epidemiological investigation and MLVA analysis, and our results indicated that the whole genome wide-SNP analysis in this case did not provide higher discriminatory power [31].

**Table 1:** Bacterial strains sequenced in this study.

MSTree ID*	Original ID	Date of Isolation	Location	Source
r	090952a	2009-07-30	Xinghai	Patient L (Before AT^#^)
	090952b	2009-07-31	Xinghai	Patient L (After AT^#^)
	090953	2009-07-31	Xinghai	Patient K
	090954	2009-07-30	Xinghai	Patient J
	090955	2009-07-26	Xinghai	Patient A (Index patient)
	090958	2009-08-05**	Xinghai	*Canis familiaris* (Dog I)
	090959	2009-08-05**	Xinghai	*Canis familiaris* (Dog II)
u	05001	1956	Xinghai	Flea in *Marmota himalayana*
s	05008	1960	Xinghai	*Marmota himalayana*
t	05006	1960	Xinghai	*Marmota himalayana*

**Figure 3:**
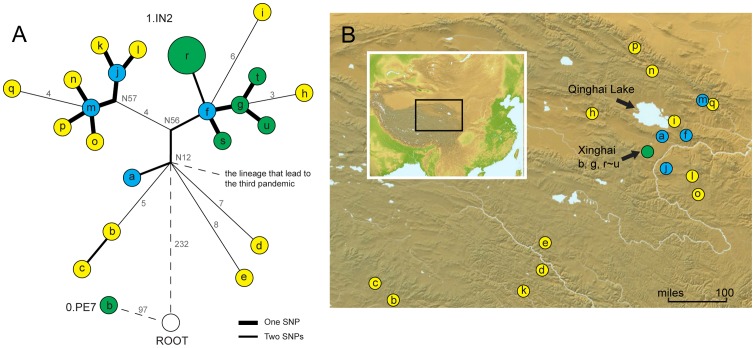
Second step of source tracing: Investigating the phylogenetic relationship within the particular phylogroup and historical isolates in the outbreak region. (A) MSTree of strains in the phylogroup 1.IN2 (a to q), 2009 outbreak strains (r), and historical strains in Xinghai County (s, t, u, and 0.PE7b). The circle size represents the number of strains it contains. The designation of hypothetical nodes was obtained from previous research [32], and the SNP numbers (>2) between the nodes are labeled in gray on each branch. Strains that were close to the MRCA of phylogroup 1.IN2 (node N12) are marked in blue, strains isolated from Xinghai are in green, and the other strains are in yellow. (B) Geographic distribution of strains used in MSTree.

: MSTree ID refers to the strain ID in Figure 3A.

#: AT stands for antibiotic treatment.

: The two dogs that died in the outbreak were buried on July 22 and 23, 2009, respectively [31]. For the epidemic investigation, the bodies were exhumed, and strains 090958 and 090959 were successfully isolated from the necropsy samples on August 5, 2009.

In the phylogenetic tree, the outbreak strains were most closely related with a strain from a previously defined phylogroup 1.IN2, with only three SNP variations observed between them [32]. Actually, 9 and 15 SNPs distinguished the outbreak strains from the strains of phylogroups 1.IN1 and 1.IN3, respectively, and more than 20 SNPs with the other phylogroups. Therefore, the outbreak strains were a clone that belongs to phylogroup 1.IN2. Seventeen strains were previously identified in 1.IN2 [32], and all these strains were distributed in the Qinghai-Tibet Plateau of China, which suggests that the outbreak strains also originated from this region. However, as the Qinghai-Tibet Plateau is very wide (around 2.5 million km^2^) and the 1.IN2 strains actually dispersed in two distinct sections in the plateau (Figure 3B, top right and bottom left), the source tracing result obtained by phylogenetic analysis of the outbreak and representative strains representing global diversities was still lack of resolution.

To trace the source of the outbreak strains with higher resolution, we employed the second step of the new strategy. Another set of genome sequences, including 17 strains of phylogroup 1.IN2 and five historical strains isolated from Xinghai, was used to construct the phylogeny with outbreak strains. All Xinghai isolates (marked by green circles in Figure 3) belong to the same subclade, except for one strain, 0.PE7b, which belongs to the currently known most ancient *Y. pestis* lineage and varies with other 1.IN2 strains by more than 300 SNPs. Notably, although the *Y. pestis* strains have been isolated from Xinghai several times since 1956, the 2009 outbreak isolates were not the direct descendant of any of these historical isolates. One strain, 1.IN2f, that was isolated in 1971 from the adjacent county, Huangyuan, was located intermediately before the outbreak strains, and only two SNPs were observed between this strain and the outbreak ones (Figure 3). Therefore, the 2009 Xinghai plague outbreak most likely resulted from the infection of *Y. pestis* strains that had survived in other counties that surround the Qinghai Lake region.

### Historical epidemiology of *Y. pestis* of a specific phylogroup

Multiple polytomies, including N12, 1.IN2f, g, j, and m, were observed in the phylogeny of phylogroup 1.IN2 (Figure 3A). The polytomy at node N12 represents the most recent common ancestor (MRCA) of all strains of the phylogroup 1.IN2 and leads to the lineage that caused the third plague pandemic. Similar to the “big bang” polytomy that was connected with the Black Death, the N12 also possibly resulted from a large-scale epidemic, which was estimated to have occurred 212 years ago (95% confidence interval between 116 and 337 years) [32]. A human plague epidemic occurred around 260 years ago in Qinghai Province (Year 1754), when an infected monk traveled around the Qinghai and Gansu Provinces to preach Buddhism, which then led to a large-scale plague outbreak [33]. No further details were available because scientific knowledge was limited during that time. Of the four out of five 1.IN2 strains that were isolated from Tibet, 1.IN2b-e radiated from the node N12, which suggests that the ancestors of these strains were transmitted to Tibet by a single ancient epidemic within a relatively short time interval. One Tibet isolate, namely, 1.IN2k, which was located in the other branch and was a direct descendent of a Qinghai isolate, 1.INj, indicates a recent independent spread event of plague pathogens from Qinghai to Tibet.

The other polytomies also suggested historical expansion of the *Y. pestis*. Interestingly, the strains that coincide with the polytomies, including 1.IN2f, j, and m, were all isolated from a small region east of Qinghai Lake. In addition, 1.IN2a, a strain that is mostly close to the MRCA of the phylogroup 1.IN2, was also isolated from this region (Figure 3B). These observations suggest that although the strains of phylogroup 1.IN2 were widely distributed in the Qinghai-Tibet Plateau, the majority of epidemics caused by this phylogroup originated from a particular region east of Qinghai Lake.

## Discussion

We used a two-step phylogenetic analysis that is based on different sets of genomes to prove that the 2009 plague outbreak originated from the infection of *Y. pestis* that circulated in a local region as well as verified the previous conclusion of dog–human transmission [31]. The first step was to define the phylogenetic position of outbreak isolates by comparing the representative strains of *Y. pestis* with worldwide ones to deduce a preliminary outbreak origin. The second step was to acquire an accurate, more distinct source tracing result by reconstructing the phylogenetic relationship among all known genomes that belong to the same phylogroup as the outbreak strains and the genomes of historical strains isolated from the outbreak region. A one-step strategy that explored all available genomes (including all phylogroups) to compare with the outbreak strain can provide a source tracing result with similar resolution. However, this process significantly increased the consumption of computational resources and, correspondingly, the analysis running time. The two-step strategy included the reconstruction of MLTree of 31 genomes and MSTree of 28 genomes based on 1,564 and 387 SNPs, respectively. This analytical strategy could be accomplished by using a laptop with regular hardware (2.7 GHz CPU, 2 GB RAM) within a 16 min running time to reconstruct the MLTree with 1,000 bootstrap iterations (the first step) and less than 1 min to reconstruct the MSTree with hypothetical nodes (the second step). In contrast, building the MLTree of all 133 known genomes and seven outbreak strains requires at least 5 h. Accordingly, the two-step strategy would significantly reduce the time cost of the data analysis step in source tracing, which is important in dealing with the outbreak of infectious disease. Additionally, the two-step strategy is more efficient when employed for an extra large dataset as it could effectively reduce the number of genomes that are being analyzed.

The two-step analytical strategy would be helpful not only in tracing the source of *Y. pestis* but also in investigating other important emerging infectious diseases as well as verifying if a bioterrorism attack indeed occurred. However, the wide application of the strategy will depend on extensive published whole genome sequences as well as on comprehensive comparative analysis across these sequences, which involves defining the representative strains for each phylogroup of a particular pathogen species and developing an international database to integrate the genome data and provide convenient analysis tools for the public. The two-step strategy requires low computational ability, which ensures that genome-based source tracing results can be generated even with a laptop. A third-generation sequencing machine, such as Ion Torrent PGM based on semiconductor technology, is able to determine the whole genome sequence within hours [34] despite the fact that it is no larger than a desktop printer. Therefore, both the sequencing and analysis machines are fast and portable. The rapid development of sequencing technology and analysis methods indicate that conducting on-site or real-time source tracing is feasible in the near future.

A small region located east of Qinghai Lake is the possible source of most of the historical epidemics caused by the strains of phylogroup 1.IN2, which suggests that the natural environment in this region is suitable for the long-term survival of *Y. pestis*. As a matter of fact, plague endemics affect marmots population in this region almost every year, and humans are often infected when they hunt and skin marmots [35]. Strains in the most ancient lineage of *Y. pestis*, 0.PE7, were also isolated from this region, which supports the hypothesis that this region is a possible source of *Y. pestis* [32]. Therefore, intensive ecological investigation on this region should be conducted to further understand the maintaining mechanisms of *Y. pestis* in its natural foci. In addition, intensive surveillance of this nest of *Y. pestis* would largely promote plague prevention and control.

## Materials and Methods

### Bacterial strain and DNA preparation

We determined the genome sequences of 10 *Y. pestis* strains in this study (Table 1), including five strains that were isolated from patients and two from dogs that died in the 2009 Xinghai primary pneumonic plague outbreak [31], and three other historical isolates (1956–1960) in the same county that were isolated from marmots and fleas. Published *Y. pestis* genome sequences [32], including 24 that could represent the global diversity of *Y. pestis* species and 17 that belong to the same phylogroup as the outbreak strains, were also analyzed in this research (Table S1).

*Y. pestis* strains were cultivated in Luria–Bertani broth at 26°C for 48 h, and DNAs were extracted and purified by using conventional sodium dodecyl sulfate lysis and phenol/chloroform extraction. DNA concentrations were quantified by using ultraviolet spectrometry.

### Sequencing and assembly

Multiplexing paired-end libraries were created with an average insert size of 500 bp for each isolate according to the manufacturer's instructions. Whole genome de novo sequencing was then performed by using Illumina GA II (Illumina Inc.). A total of 98 million reads of 75 bp length, which equal 7,279 MB of raw data, were generated for all of the 10 genomes. For each isolate, raw short-read sequences were subjected to de novo assembly by using SOAPdenovo [36]. All raw reads were remapped onto the contigs by using SOAPaligner [37]. The average effective sequencing depth for all isolates is 119-fold, with more than 74 million effective reads that cover over 99.88% of the assembled sequences (Table S2), which indicates that the assemblies are reliable. The raw reads were deposited at GenBank under accession number SRA010093.

### SNP calling and validation

SNPs were identified through a pairwise comparison of the contigs of the 10 genomes by using the MUMmer package [38]. MUMmer results were accumulated, and the unreliable SNPs, which were located in repeat regions, had low base quality (quality scores of <20), or were supported by few reads (<10 pair-end reads), were filtered out as described earlier [32]. Only five novel SNPs were identified in addition to the 2,298 previously defined ones of *Y. pestis* [32]. The alleles of five novel SNPs are presented in the reference genome (strain CO92, accession number: NC_003143), and their annotation information were listed in Table S3. The newly identified SNPs were validated by polymerase chain reaction–Sanger sequencing by using primers listed in Table S4.

### Phylogenetic analysis

The genome of *Y. pseudotuberculosis* strain IP32953 (accession number: NC_006155), was employed as the outgroup in this research. The nucleotide in each SNP locus, which is consistent with the allele in IP32953 genome, was designated as the parental status, whereas the inconsistent one was designated as the mutational status. The maximum likelihood tree (MLTree) was constructed based on concatenated SNPs by using the software PHYML [39]. The neighbor-joining and the maximum parsimony trees based on the same SNP set were separately constructed by using MEGA 5.1 with 1,000 bootstrap iterations [40]. The minimal spanning tree (MSTree) was constructed by using BioNumerics 5.1 (Applied Maths, Belgium), which allows the creation of hypothetical nodes. There are 1,564 informative SNPs presented in 24 representative strains and seven outbreak strains, which was used in construction the phylogeny in the first step; and 348 informative SNPs in 17 strains of 1.IN2 phylogroup, five historical strains isolated from Xinghai, and the seven outbreak strains were used to construct the phylogeny in the second step.

### Computational cost estimation by using simulated mega dataset

We created 46 simulation datasets that included 500 to 5,000 genome sequences for each, with 100 genomes increasing progressively. As the smallest dataset was set to include 2,664 SNPs in 500 genomes and one more genome will take one novel SNP into the dataset, the largest simulated dataset contained 7,164 SNPs in 5,000 genomes. We constructed MLTrees for each dataset based on concatenated SNPs by using RAxML v7.2.6 with a substitution model of GTR GAMMA [41]. The software ran under the CentOS v5.5 operating system. The CPU running time and random access memory (RAM) consumption for each run were recorded separately.

## Supporting Information

Figure S1 **Phylogenetic tree of outbreak strains and representative strains with global diversity that are based on maximum parsimony (A) and neighbor-joining (B) method.** The branches of 0.PE3 are truncated in both panels A and B because of their unusual length. The nodes with less than 90% bootstrap support were indicated in the trees.
(TIF)

Table S1 ***Y. pestis*** **strains sequenced in previously research.**
(XLS)

Table S2 **Data production of the strains sequenced in this research.**
(XLS)

Table S3 **List of SNPs used in phylogenetic analysis.**
(XLS)

Table S4 **Primers using in SNPs validation.**
(DOC)
