# Supplementary figures and images for: Two-Step Source Tracing Strategy of Yersinia pestis and Its Historical Epidemiology in a Specific Region
Source: PLoS One. 2014 Jan 9;9(1):e85374. doi: 10.1371/journal.pone.0085374 (PMC3887043; doi:10.1371/journal.pone.0085374)

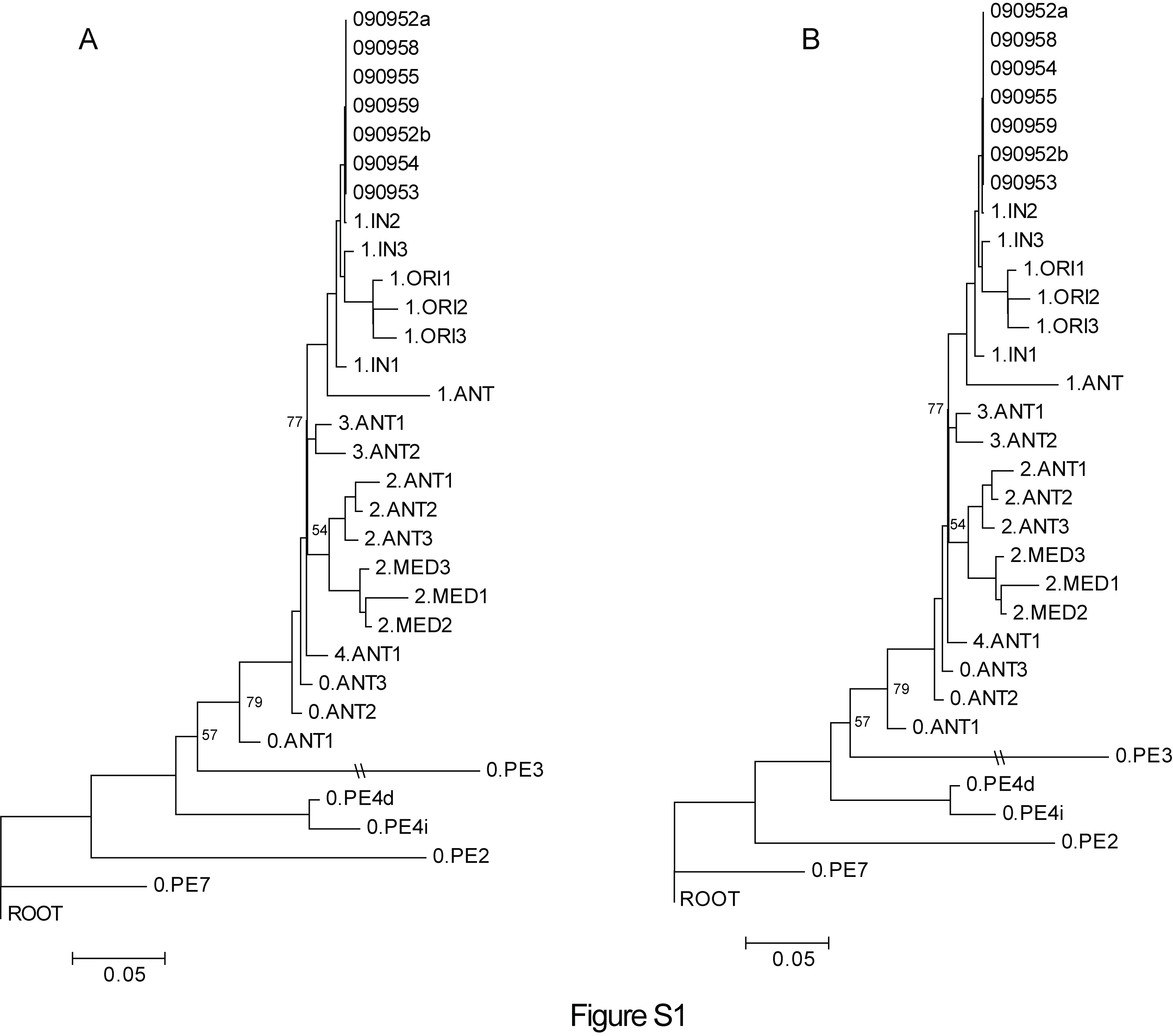

Supplement: Figure S1 — Phylogenetic tree of outbreak strains and representative strains with global diversity that are based on maximum parsimony (A) and neighbor-joining (B) method. The branches of 0.PE3 are truncated in both panels A and B because of their unusual length. The nodes with less than 90% bootstrap support were indicated in the trees. (TIF) [file pone.0085374.s001.tif]
