# Supplementary material for: Two-Step Source Tracing Strategy of Yersinia pestis and Its Historical Epidemiology in a Specific Region
Source: PLoS One. 2014 Jan 9;9(1):e85374. doi: 10.1371/journal.pone.0085374 (PMC3887043; doi:10.1371/journal.pone.0085374)
Supplement: Table S4 — Primers using in SNPs validation. (DOC) [file pone.0085374.s005.doc]

**Supplementary Table 4. Primers using in SNPs validation**

| **SNP ID** | **Primer ID** | **Primer sequences (5’-3’)** |
| --- | --- | --- |
| s3727 | s3727-F | GCAACCTTGGTGAAGATCAATG |
|  | s3727-R | CGCCTCAATCATCTGGATACTG |
| s3728 | s3728-F | GACAGCAATACCATCGCCATC |
|  | s3728-R | TGCCGTAAACCGCTCCTG |
| s3729 | s3729-F | CGTTGTCACCTGTGGATTTGG |
|  | s3729-R | CCTGCTTATCACCTTGCTGTTC |
| s3730 | s3730-F | TGCCGCCTCTGGAGAATG |
|  | s3730-R | GGAGCAACCGTTAGTCTGG |
| s3731 | s3731-F | TTGATGCTCTGCCGTCTATTG |
|  | s3731-R | GCTCCAGTGTTTCGCCATC |
